# Keep on Learning: Academic exhaustion and teaching evaluation in digital times

**DOI:** 10.1007/s41358-021-00294-z

**Published:** 2021-11-04

**Authors:** Sandra Brunsbach, Ralph Kattenbach, Ines Weber

**Affiliations:** 1 Institute of Social Sciences, Department of Political Science, Christian-Albrechts-Universität zu Kiel, Kiel, Germany; 2 International School of Management ISM, Hamburg, Germany

**Keywords:** Online teaching, Higher education, Evaluation, Academic exhaustion, Online-Lehre, Hochschulbildung, Evaluation, Akademische Erschöpfung

## Abstract

Since the onset of the Corona pandemic in early 2020, teaching and learning at universities has changed profoundly due to the measures aimed at contact reduction. The present study aims to evaluate online teaching in political science from a students’ perspective. Based on a quantitative online survey, an evaluation of sub-facets of online teaching compared with classroom teaching has been measured.

Moreover, three impact factors on the global evaluation level have been identified and tested. Study constraints are supposed to influence online teaching evaluation negatively. Dialogue with lecturers as well as social exchange with fellow students are assumed to have a favourable effect. A mediating role of academic exhaustion is hypothesised to explain the effect of these factors on teaching evaluations both for online lectures and online seminars.

Our data support the assumed negative effect of study constraints on teaching evaluation and a positive effect of social exchange. Dialogue with lecturers has no significant impact. While the relationship between study constraints and evaluation is fully mediated, the impact of social exchange is partly mediated by academic exhaustion. These interrelationships are evident for both online lectures and online seminars. Practical implications for future teaching in political science are discussed.

## Abstract (de)

Seit dem Ausbruch der Corona-Pandemie Anfang 2020 hat sich das Lehren und Lernen an den Hochschulen durch die Maßnahmen zur Kontaktreduzierung grundlegend verändert. Die vorliegende Studie zielt darauf ab, die Online-Lehre in der Politikwissenschaft aus der Perspektive der Studierenden zu evaluieren. Basierend auf einer quantitativen Online-Befragung wurde eine Bewertung von Teilbereichen der Online-Lehre im Vergleich zur Präsenzlehre vorgenommen.

Darüber hinaus wurden drei Einflussfaktoren auf einer generellen Bewertungsebene identifiziert und getestet. Es wird davon ausgegangen, dass Restriktionen hinsichtlich der Studienvoraussetzungen die Bewertung der Online-Lehre negativ beeinflussen. Der Dialog mit den Dozierenden sowie der soziale Austausch mit anderen Studierenden sollen sich günstig auswirken. Eine mediierende Rolle der akademischen Erschöpfung wird angenommen, um den Effekt dieser Faktoren auf die Lehrevaluation sowohl für Online-Vorlesungen als auch für Online-Seminare zu erklären.

Unsere Daten stützen den angenommenen negativen Effekt von eingeschränkten Studienvoraussetzungen auf die Lehrevaluation und einen positiven Effekt des sozialen Austauschs. Der Dialog mit den Dozierenden hat keinen signifikanten Einfluss. Während der Zusammenhang zwischen Studienbeschränkungen und Bewertung vollständig mediiert ist, wird der Einfluss des sozialen Austauschs teilweise durch akademische Erschöpfung mediiert. Diese Zusammenhänge sind sowohl bei Online-Vorlesungen als auch bei Online-Seminaren zu beobachten. Es werden praktische Implikationen für die zukünftige Lehre in der Politikwissenschaft diskutiert.

## Introduction

With increasing and improving technical solutions for online teaching in the last two decades, there has been a growing discussion, particularly in the Anglo-American world, regarding the advantages and disadvantages of online courses in higher education (Hiltz and Turoff 2005; Dumford and Miller 2018). However, with the onset of covid-19 in early 2020, online teaching experienced a revolutionary boost, and it will most probably continue to shape the university landscape and teaching in the years following the pandemic.

Teaching and learning at universities have changed profoundly due to the measures aimed at contact reduction. The ad hoc changes in teaching that have become necessary in the pandemic placed particular demands on lecturers and students. On the part of the lecturers, these include extended working hours, a more difficult reconciliation of family and career (Brunsbach and Weber 2020) and the (further) development of digital teaching and examination formats (Klonschinski et al. 2020), as well as even greater consideration of the life situations of students. For students, the pandemic measures often have clear consequences regarding their lifestyles, financial resources and mental health. At the same time, they are confronted with the digital continuation of their studies. This situation requires a high degree of adaptability and stress resistance and increases the likelihood of academic exhaustion to occur.

While the summer term 2020 was characterised by emergency remote teaching, as it was necessary to build up the required infrastructures and to develop and implement new teaching formats ad hoc (Hodges et al. 2020; Iglesias-Pradas et al. 2021), some routine set in from the winter semester 2020/2021 onwards. During the summer, universities offered didactic and technical training, lecturers had time to adapt their teaching formats, learning objectives and didactic tools, and students got initial experiences with online teaching. Even though students are still affected and burdened by the pandemic measures in their personal lives, they have had time to get used to this ‘new normal’. Against this background, we aimed to take stock of online political science teaching at the end of the winter semester 2020/2021.

Our study aimed at two objectives. First, collecting students’ online teaching evaluations when online teaching at universities has already become somewhat practised. In order to obtain a detailed and at the same time compact result, we address different facets regarding online teaching in comparison to face-to-face teaching as well as a general assessment. Second, we analyse influencing factors such as study constraints, communicative resources, and academic exhaustion on the overall evaluation. These are known to influence teaching evaluations generally and are even more relevant under pandemic conditions. For the study design, we draw on existing findings regarding online teaching, especially in political science, and on the situation of students during the covid-19 pandemic.

In the following, we provide findings regarding existing online teaching evaluations under pandemic conditions. In particular, we highlight how the study design and research question influence the results to draw conclusions for the present study. Subsequently, impact factors regarding the evaluation of online courses will be discussed to derive our hypotheses. In the method section, our online survey among political science students from Kiel University is described. Study results are presented and discussed in the following chapters.

## Evaluation of online teaching

### Evaluation of online teaching in higher education

During the pandemic online teaching is delivered in various ways, both in seminars and lectures. Examples include the provision of podcasts, videos or worksheets and the usage of learning platforms, forums and chats. However, the proportion of synchronous teaching organised through video conferencing predominates at European universities (Doolan et al. 2021, p. 13; Becker et al. 2020, p. 687; Aristovnik et al. 2020, p. 8).

How well digital university teaching works in the pandemic is difficult to answer because so many different perspectives can be taken on this question. First, the perception of lecturers and students varies, with lecturers generally painting a somewhat more positive picture (Becker et al. 2020; Universität Potsdam 2020).

Second, the type of question is decisive. Different evaluations emerge depending on whether overall satisfaction is surveyed or single facets of the teaching and learning experience are assessed. While online teaching is generally judged to be principally successful (Cicha et al. 2021, p. 13 f.), students and lecturers see the advantages and shortcomings of online learning and teaching. The perceived advantages include greater flexibility and time saving due to the elimination of travel times (Breitenbach 2021, p. 10; Doolan et al. 2021, p. 43). Disadvantages, on the other hand, are seen in a more inferior quality of discussion within seminars, difficulties for students to concentrate and perceived isolation in the learning process (Becker et al. 2020; Aguilera-Hermida 2020; Mishra et al. 2020; Doolan et al. 2021, p. 44; Jeffery and Bauer 2020; Eberle and Hobrecht 2021; Bork-Hüffer et al. 2021, p. 9).

Third, differences occur according to the type of course. The digital implementation of lectures is rated better than that of seminars or practical exercises (Drašler et al. 2021, p. 9). The most common division in terms of the type of online teaching is that of synchronous and asynchronous forms. While some studies conclude that students prefer synchronous online classes (Doolan et al. 2021, p. 48), others show a divided picture (Becker et al. 2020, p. 692).

Fourth, results are worse in some evaluation categories when an explicit comparison is made with face-to-face teaching. Such comparisons have already been made before the pandemic started. Especially in the Anglo-American world, online study programmes have already been widely used, and the success of these programmes has been compared to traditional classroom teaching. Many studies show that the learning success of students also of political science measured by examination results, subject knowledge or interest in the subject matter is equally high in online courses and face-to-face courses (Ni 2018; Nygren 2015; Stack 2015; Soffer and Nachmias 2018; McPhee and Söderström 2012; Roscoe 2012). The same applies to hybrid courses (Bolsen et al. 2016). Nevertheless, the course completion and the retention rate show that online courses are less successful. Dropping out is significantly higher for students in an online degree programme (Glazier et al. 2021; Hamann et al. 2020; Hart et al. 2018). Reasons are, among others, the socio-demographic characteristics of the students and their social environment, which differ significantly from that of other students, as well as the class design and the often less favourable instructor-student interaction in online courses (Glazier 2016, p. 438 ff.; Lee and Choi 2011). These findings are free of Corona pandemic effects on students’ well-being and life situations, and the evaluations deal with conceptually prepared online classes instead of emergency ad-hoc implementations. However, the disadvantage of this perspective is that participants only know one of the two teaching formats. The comparative evaluation in the pandemic records the assessment of people who have experienced both teaching formats. There are only a few studies that make such a direct comparison for teaching in times of Covid-19. For lecturers, Becker et al. (2020) find that two-thirds consider the quality of face-to-face teaching to be superior to online teaching. A survey of students by Aguilera-Hermida (2020) also indicates a preference for face-to-face teaching. Drašler et al. (2021) show a similar assessment for laboratory exercises, but not for lectures, where students are pretty open to digital implementation. However, when asked about various aspects of the learning process, such as the acquisition of knowledge, interaction with lecturers and the knowledge assessment, both students and lecturers prefer face-to-face teaching (Drašler et al. 2021, p. 9).

In conclusion, the students’ perspective fits our interest in their perceptions and their (study) life situation. Evaluating single facets is more detailed and provides information for future improvement. Since the lecture and seminar formats differ significantly, we suspect diverging implementation successes in online teaching. As evaluations are always based on implicit comparisons, we provide a reference framework to compare online teaching with classroom teaching. In this way, we set a benchmark that makes the results easier to interpret. Finally, we opted for an online survey at the end of the 2nd semester to capture influences free of the emergency situation in the first pandemic semester. All considerations taken together, we decided to conduct a student evaluation (1) of individual facets (2) separately for lectures and seminars/tutorials (3) in comparison to face-to-face teaching before Covid-19 (4).

### Impact factors on the evaluation of online teaching

Beyond evaluating individual facets, identifying impact factors responsible for interindividual differences in evaluations provides essential information to improve online teaching further. Considering the existing literature on the teaching-learning process at universities and the specific situation of students during the pandemic three factors appear to be of particular importance: a) the general life situation of students and the associated individual conditions under which they study, b) the extent of communication and dialogue with lecturers and c) the exchange with other students.

Concerning students’ life situations during the pandemic, an apparent increase in psychological problems among students is evident in various countries. Students experience frustration, anger, hopelessness and a general deterioration in their mental well-being (Aristovnik et al. 2020, p. 15; Padrón et al. 2021; Onwuegbuzie et al. 2020; Browning et al. 2021). They worry about their financial situation, care responsibilities, and professional future (Breitenbach 2021, p. 9; Feucht et al. 2020, p. 113; Universität Potsdam 2020, p. 9; Aguilera-Hermida 2020, p. 5; Doolan et al. 2021, p. 29 f.). Of course, teaching evaluation is influenced by these circumstances. Furthermore, prerequisites like an appropriate home-office workplace and the necessary technical equipment are essential. Bork-Hüffer et al. (2021, p. 14 f.) show that living situations influence the online teaching experience. Students who lived in shared flats and thus generally had a less quiet home-office workplace showed significantly lower satisfaction with university-based online teaching (Bork-Hüffer et al. 2021). Regarding the necessary technical equipment, it appears that a large proportion of European students (around 90%) are equipped with a computer or laptop (Aristovnik et al. 2020; Doolan et al. 2021, p. 4) and can at least follow asynchronous online teaching. The situation is less suitable for synchronous online teaching because many students do not have a sufficient internet connection (Feucht et al. 2020, p. 108; Doolan et al. 2021, p. 4). We understand constricted study conditions like an inadequate workspace, unstable internet, insufficient technical equipment or even financial worries as a study demand that costs energy to cope with and therefore negatively affects the evaluation of online teaching.

#### H1:

*Constricted study conditions are negatively correlated with the evaluation of online lectures and seminars.*

Existing studies show that students suffer from isolation during the pandemic and complain about decreasing or insufficient contact with teaching staff and fellow students (Feucht et al. 2020; Breitenbach 2021).

The reduced contact with lecturers should impact evaluation, as “effective teaching is not simply delivering content” but incorporates interaction and meaningful relationship between students and lecturers (Glazier 2021, p. 175). Contact and dialogue can be considered the essential prerequisite for a student-instructor-rapport to develop. When such a relationship develops, it positively impacts student engagement, grades, and overall academic success and reduces dropout (Legg and Wilson 2009; Demir et al. 2019). This has been demonstrated for face-to-face teaching, forms of blended learning and online teaching alike (Glazier 2016; Shaw et al. 2015). In addition, some studies show a direct correlation with the evaluation of a course. The better the perceived rapport with the lecturer, the better the students’ course evaluation (Schriver and Harr Kulynych 2021). Therefore, we assume an influence of perceived contact and dialogue with lecturers on online teaching evaluation.

#### H2:

*The more dialogue there is with lecturers, the better online lectures and seminars are evaluated.*

While a professional exchange primarily characterises the relationship between lecturers and students, the relationship with other students is naturally more complex. Concerning the social exchange between students, one can distinguish on-topic communication, in which the subject-related exchange is in the foreground, and off-topic communication, in which all kinds of topics are discussed that are not directly related to the content of the degree programme. Both on-topic and off-topic exchanges with other students should impact the evaluation of online teaching. On-topic communication is closely related to peer learning, where students gain knowledge through working and discussing with other students. The positive effect of this form of student exchange on their academic performance and motivation is well documented (Núñez-Andrés et al. 2021; Nortcliffe et al. 2019; Hamann et al. 2009). Especially in political science, where lively seminar discussions have always been an inherent part of the teaching methodology, lecturers aim to build up a community of peers who actively listen to each other to increase cognitive presence and thus learning success (Roberts 2021, p. 184). Social exchange with other students that is not directly related to a subject is also relevant as it prevents loneliness, improves well-being and reduces perceived exhaustion. This should also have an impact on the evaluation of online higher education. Taken together, we expect an influence of social exchange with other students on teaching evaluations.

#### H3:

*The more social exchange with fellow students, the better online lectures and seminars are evaluated.*

We expect an impact of the beforementioned factors for seminars and lectures but simultaneously assume that the impact is more substantial for seminar evaluation. Seminars are more interactive and challenging. Students should participate and actively deal with the respective subject area. Although active learning is also being pushed more strongly for political science lectures recently (Rehder et al. 2019), these are characterised mainly by passive knowledge absorption. In times of online teaching, they are also less demanding in terms of study conditions, as they are often taught asynchronously and require no camera or microphone.

Another relevant construct for the evaluation of online teaching is academic exhaustion. Based on the two-dimensional burnout structure by Demerouti et al. (2003), exhaustion together with disengagement can lead to burnout. Academic exhaustion is a specific form of exhaustion experienced by students in the course of their learning process. They are pessimistic and feel tired due to the demands and requirements of studying. As shown below, exhaustion is related to both our impact factors and outcome variables comparable to online teaching evaluation leading us to assume a mediating role for the relationship between the hypothesised impact factors and online teaching evaluation.

Life stress and worries, e.g. about the future, are predictors of academic exhaustion (Lin and Huang 2014; Huang and Lin 2010). Particularly relevant for online teaching in a pandemic, Lin and Huang (2012, p. 239) show that loneliness strongly impacts academic burnout and thus academic exhaustion. According to the conservation of resources theory (Hobfoll 1989) and social support resource theory (Hobfoll et al. 1990), social support is a resource in dealing with stressful situations reducing the resulting experience of exhaustion. In this vein, study resources correlate negatively with exhaustion among students (Gusy et al. 2016, p. 51). In a longitudinal study, Hoferichter and Raufelder (2021) show that academic learning support from teachers reduces exhaustion among students. Mokgele and Rothmann (2014) report academic support as the most substantial study-related resource. This is a challenge for online teaching as the dialogue with lecturers comes about quickly and easily outside lesson time in face-to-face teaching but not in a virtual setting with abruptly ending sessions. Apart from academic support, social exchange with fellow students can provide social support in formal as well as in informal settings (Boud 2014), e.g. by developing self-regulation skills (Räisänen et al. 2020) and self-monitoring (de Backer et al. 2015). Therefore we suppose the study constraints mentioned above to increase academic exhaustion. Contrary, dialogue with lecturers and social exchange are assumed to decrease perceived exhaustion among students.

Several mediating roles are reported within the context of exhaustion relating to work and study. Exhaustion generally mediates the relationship between demands and absenteeism (Freund et al. 2012), and more specific, exhaustion mediates the impact of time pressure on students’ performance (Gusy et al. 2021). Exhaustion also mediates various effects on satisfaction in all kinds of professional settings (Dahri and Hamid 2018; Bailenson 2021; Sesen et al. 2011; Dodanwala and Shrestha 2021). As satisfaction can be considered a similar concept to evaluation, we assume that academic exhaustion mediates the impact of study conditions on online teaching evaluation. In other words, the worse the study situation, the more exhausted students feel, which is reflected in a negative evaluation.

#### H4:

*Academic exhaustion mediates the effects from constricted study conditions (a), dialogue (b), and social exchange (c) on the evaluation of online lectures.*

#### H5:

*Academic exhaustion mediates the effects from constricted study conditions (a), dialogue (b), and social exchange (c) on the evaluation of online seminars.*

## Methods

### Sample description

At the time of the survey, political science courses at Kiel University had been held exclusively in the form of online classes for two semesters. As early as spring 2020, many training courses were offered on how to use various learning platforms, communication tools and other online applications. In addition, there were didactic training opportunities on topics such as knowledge transfer in the digital age, the conception of online seminars or the production of teaching videos. Beyond that, there was a lively formal and informal exchange between lecturers from the political science department regarding the knowledge they had acquired, the experience they had already gained and promising new approaches in online teaching. Compared to the spring semester 2020, when a transfer to the digital space had to take place within a few weeks, we assume first routines and best practices to be established in winter 2020/2021.

In February 2021, all Kiel University students enrolled in a degree programme with a political science component were invited by email notification to participate in the online survey. Two reminders were sent. 2079 students received an invitation, 509 (response rate 24%) responded with survey participation, out of which N = 460 cases were suitable for analysis.

48.0% of the respondents identified as “female”, 44.8% as “male” and 0.2% as “inter/diverse/non-binary”. 7.0% opted for “no indication” or refused to answer. More than two-third (68.9%) of the respondents follow courses in a bachelor programme; the others follow courses in a master programme. The median age for bachelor students is between 21 and 22 years, for master students between 23 and 24 years.

### Operationalisation

#### Study constraints

The absence of eight prerequisites for online teaching was queried (0: given, 1: not given). These included stable internet, technical equipment, mastery of digital tools, knowledge in literature search, appropriate workplace, sufficient time, sufficient financial resources, and being free from worries to focus on studying. Responses were added together and indicated by their mean value.

#### Dialogue with lecturers

Participants had to indicate how sufficient they perceived the contact with lecturers in online teaching. Responses were given on a single item measure ranging from 1: not sufficient at all to 5: fully sufficient.

#### Social exchange with fellow students

The construct encompasses on-topic and off-topic exchange. Similar to the previous measure, students were asked on a single item about the sufficiency of social and professional exchange with fellow students (1: not sufficient at all to 5: fully sufficient).

#### Exhaustion

We assessed students’ exhaustion using four items (one reversed item) from the Oldenburg Academic Inventory (OLBI-S) on academic exhaustion (Reis et al. 2015). The item wording was slightly adapted to the situation in a digital semester (e.g. “After a virtual study day, I often need longer recovery times.”). Participants responded on a scale ranging from 1: strongly disagree to 4: strongly agree. Cronbach’s α = 0.84.

#### Evaluation of online teaching

In order to evaluate online teaching, respondents were asked to compare online lectures and seminars/tutorials with classroom teaching. They were asked separately for lectures and seminars/tutorials “… which teaching format performs better in terms of …” different aspects of a good teaching experience. A slide bar (ranging from 0 to 100) was used to differentiate between the absolute superiority of one teaching format and a balanced judgement. Compared to ranking scales, slide bars are characterised by provoking a gut feeling response, which was the intention here. For lectures, participants evaluated their concentration and attention, feeling of having learned something, and sensing their own learning level. For seminars/tutorials, the facets included regular attendance, active participation, concentration and attention, the feeling of having learned something, discussion quality, and sensing one’s own learning level. The evaluation of single aspects reflects an underlying global judgement towards online teaching as indicated by a high internal consistency (Cronbach’s α = 0.85 for lectures and 0.87 for seminars and tutorials). Therefore, the general evaluation of the online lectures or seminars is given by the mean value of the respective items mentioned above.

#### Control variables

Age is measured initially on an ordinal scale from < 20 years to > 33 years. For regression analyses, a dichotomous variable Age > 24 is calculated. We assume increased autonomy and self-responsibility for the older group. For instance, parents in Germany receive child allowance for their studying kids up to this age level, which is often passed on. A second control variable is gender, which is also dichotomised to enter metric analysis procedures.

## Analysis

**Fig. 1:**
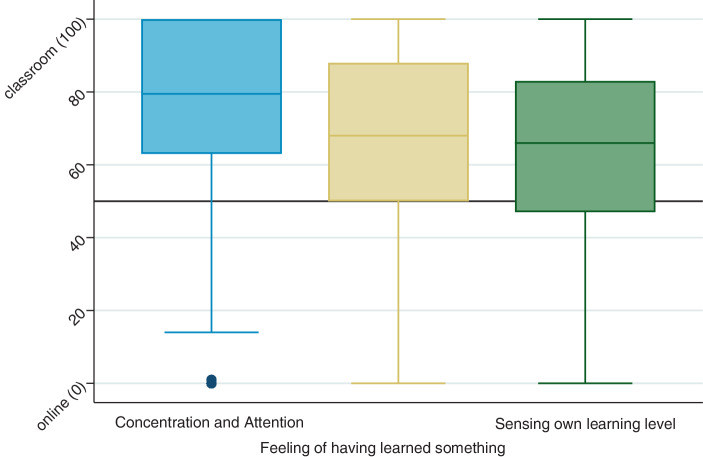
Online vs Face-to-Face—Lectures

Students who attended lectures in both the traditional face-to-face form and as online lectures were asked to indicate which variant they preferred concerning various aspects. Fig. 1 shows the boxplots for the response distributions (*n* = 249). In all three aspects, there is a clear preference for face-to-face lectures. The median for all aspects is above 50 (concentration and attention (79.5), feeling of having learned something (68.0), sensing own learning level (66.0)) and thus within the scale range where respondents tend more towards face-to-face lectures than online lectures. The interquartile range is also clearly in this range, except for the feeling of one’s own learning level. At the same time, both the median and the dispersion of the values show a varying degree of preference for classroom teaching. This is strongest for concentration and attention and lowest for the perception of one’s learning level. Concerning the latter facet, more than 25% of the students prefer online lectures.

**Fig. 2:**
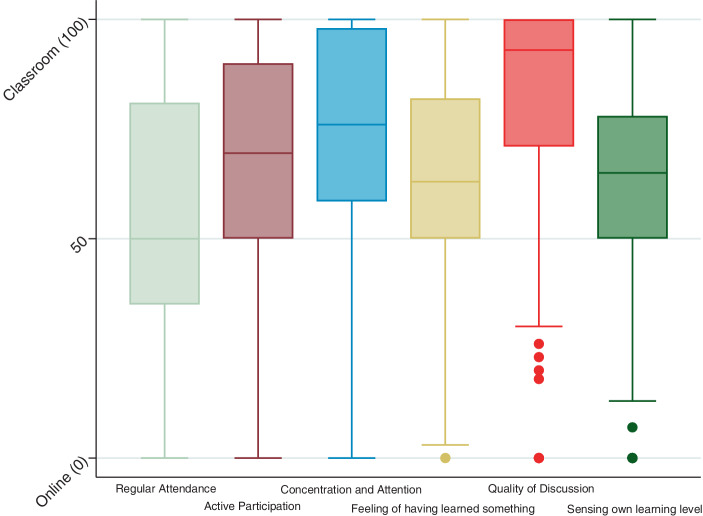
Online vs Face-to-Face—Seminars

Fig. 2 shows the students’ preference for seminars as a teaching format (*n* = 222–242). Here, too, there is a general preference for face-to-face seminars. However, the aspect of regular attendance is an exception. Here the median is precisely 50, and neither a preference for face-to-face teaching nor the online variant is indicated. Twenty-five per cent of the students tend to prefer the online version here slightly (the lower quartile limit is 35), and another 25% see a clear superiority of online seminars for this aspect. For all other facets surveyed, both the median and the interquartile range are above 50 and thus in the range that indicates a preference for face-to-face teaching. This preference is most pronounced with regard to concentration and attention (median 76.0) and discussion quality (median 93.0). Comparing the facets of concentration and attention, the feeling of having learned something, and the sensing of one’s own learning level, it becomes apparent that the preference for face-to-face teaching in these areas is marginally less strong for seminars than for lectures.

Correlations and descriptive statistics are displayed in Table 1. Note that the number of responses for the lecture and seminar evaluation is half as large as for the other variables due to the measurement design in which online teaching was compared to classroom teaching. The large group of first-year students could not respond to these questions due to a lack of experience with classroom teaching.

**Table 1:** Correlation matrix

	N	M (SD)	1	2	3	4	5	6	7
1 Exhaustion	458	2.73 (0.98)	1	–	–	–	–	–	–
2 Study Constraints	441	−0.66 (0.22)	0.43***	1	–	–	–	–	–
3 Dialogue	428	3.16 (1.18)	−0.27***	−0.17***	1	–	–	–	–
4 Social exchange	443	2.02 (1.17)	−0.31***	−0.18***	0.33***	1	–	–	–
5 Lecture evaluation	268	68.23 (22.87)	0.34***	0.12*	−0.07	−0.23***	1	–	–
6 Seminar evaluation	243	67.39 (20.07)	0.46***	0.16*	−0.18**	−0.38***	0.71***	1	–
7 Age > 24	436	0.25 (0.44)	−0.05	0.10*	0.04	−0.09	−0.13*	−0.17**	1
8 Gender	427	0.52 (0.50)	0.18***	0.25***	−0.00	0.04	0.04	−0.02	−0.09

*N* sample size, *M* mean value, *SD* Standard deviation

*p < 0.05; **p < 0.01; ***p < 0.001 (2-tailed significance)

Academic exhaustion shows significant relationships with all other variables except age. For online teaching evaluations, the correlations are among the strongest. The more exhausted students are, the worse their evaluation is in comparison to classroom teaching. This holds true for lectures (*r* = 0.34; *p* < 0.001) and even more for the more interactive formats of seminars and tutorials (*r* = 0.46; *p* < 0.001). In other words, exhaustion alone can explain 13% of the variance in online lecture evaluations and 23% of the variance in online seminars and tutorials evaluations. Note that with increasing value, online evaluations are less favourable compared to classroom teaching. Constricted study conditions also have significant relationships with all other variables, including age.

Table 2 shows the results of stepwise multiple regression analyses for academic exhaustion, online lecture evaluation and online seminar evaluation. Control variables and study constraints entered the first model. The communicative resources dialogue and social exchange are included in Model 2.

**Table 2:** Hierarchical regressions

	Academic Exhaustion		Lecture Evaluation		Seminar Evaluation	
	Model 1	Model 2	Model 1	Model 2	Model 1	Model 2
*Controls*						
Age > 24	−0.09*	−0.12**	−0.14*	−0.13*	−0.19**	−0.20**
Gender	0.06	0.07	−0.01	−0.02	−0.10	−0.06
Study constraints	0.43***	0.37***	0.15*	0.11	0.21**	0.16*
*Social resources*						
Dialogue	–	−0.13**	–	0.02	–	−0.03
Social exchange	–	−0.24***	–	−0.24***	–	−0.36***
*R*^*2*^	0.21***	0.30***	0.04*	0.08**	0.07***	0.21***
*Adj. R*^*2*^	0.20	0.29	0.03	0.06	0.06	0.19
*ΔR*^*2*^	–	0.09***	–	0.05**	–	0.14***
*N*	424	388	255	235	237	211

stand. coefficients ß displayed

*p < 0.05, **p < 0.01, *** *p* < 0.001

Analysing the antecedents of academic exhaustion, our stepwise regression model (Table 2) explains 30% of the total variance. Study constraints represent the strongest impact factor (*ß* = −0.37; *p* < 0.001). The more constraints there are for a trouble-free study, the more exhausted the students are. Concerning the effect of communication, both the dialogue with lecturers (*ß* = −0.13; *p* < 0.001) and even more the exchange with fellow students (*ß* = −0.24; *p* < 0.001) reduce exhaustion in line with our assumptions. The significant bivariate correlation of gender disappeared; in turn, age becomes a significant but still weak regressor in the multivariate model. Students older than 24 are slightly less exhausted then their younger fellow students (*ß* = −0.12; *p* < 0.01).

The regression model only marginally explains the variance in evaluations of online lectures. Study constraints lead students to prefer face-to-face lectures more strongly (*ß* = 0.11; *p* < 0.05) while social exchange has a positive effect on online-lecture evaluation (*ß* = −0.24; *p* < 0.001). The dialogue with lecturers remains insignificant. Older students prefer face-face lectures less strongly than their younger fellow students (*ß* = −0.13; *p* < 0.05).

The regression model for seminar evaluation has a better fit. 22% of the variance in the evaluation is explained by the model. Study constraints remain a significant factor in model 2 (*ß* = 0.16; *p* < 0.05). Online seminar evaluation is less favourable with increasing constraints. Among the communicative resources again, only social exchange with fellow students has a significant positive effect which is more than twice as strong as the effect of constraints (*ß* = −0.36; *p* < 0.001). Again, there is no gender but an age effect. Older students give more positive evaluations for online seminars (*ß* = −0.20; *p* < 0.01).

Hypothesis 1 on the impact of constrained study conditions is supported for seminar and lecture evaluation. Hypothesis 2 on the impact of dialogue with lecturers has to be rejected. Hypothesis 3 on the positive influence of social exchange gets empirical support for lecture and seminar evaluation.

Finally, we tested the mediating role of exhaustion on the effect of demanding study constraints and communicative resources on online evaluations based on PROCESS Model 4 by Hayes (2018), which uses ordinary least squares regression, yielding unstandardised path coefficients for total, direct, and indirect effects. Bootstrapping with 5000 samples were employed to compute the confidence intervals and inferential statistics. Effects were deemed significant when the confidence interval did not include zero.

**Fig. 3:**
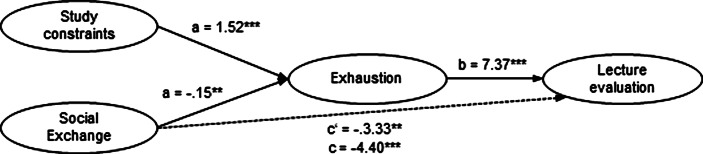
Mediation model for lecture evaluation. *N* = 244; ***p < 0.001; unst. coefficients B; c: total effect; c′: direct effect; a*b: indirect effect

For online lecture evaluation as a dependent variable (Fig. 3), two mediations with covariates were performed to analyse whether the direct paths from social exchange and study constraints would be mediated by academic exhaustion. After entering the mediator into the model, social exchange predicts exhaustion significantly, a = −0.15; *p* < 0.01, the same is true for study constraints a = 1.52; *p* < 0.001. Exhaustion, in turn, predicts the evaluation of online lectures significantly, b = 7.37; *p* = 0.001. The relationship between study constraints and lecture evaluation is fully mediated by academic exhaustion (1.07, 95%–CI [−2.10; −0.25]), the relationship between social exchange and lecture evaluation is partly mediated (*ab* = 11.22, 95%-CI [5.87; 17.65]).

**Fig. 4:**
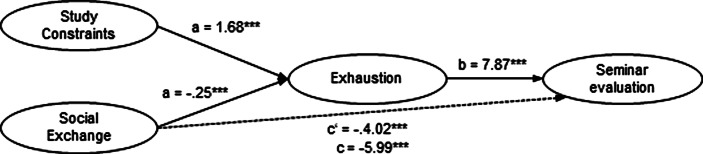
Mediation model for seminar evaluation. *N* = 244; ***p < 0.001; unst. coefficients B; c: total effect; c′: direct effect; a*b: indirect effect

For online seminar evaluation as a dependent variable (Fig. 4), two mediations with covariates were performed as we assumed a mediating effect of exhaustion on the direct paths of two independent variables, study constraints and social exchange. Study constraints and social exchange predict exhaustion significantly (a_1_ = 1.68, *p* < 0.001; a_2_ = −0.25; *p* < 0.001), which in turn predicts the evaluation of online lectures significantly, b = 7.87; *p* = 0.001. The relationship between study constraints and seminar evaluation is fully mediated by academic exhaustion (*ab* = 13.27, 95%-CI [8.14, 19.11]), the relationship between social exchange and seminar evaluation is partly mediated (*ab* = −1.98, 95%-CI [−3.11, −1.02]).

The mediation hypotheses are supported for all significant direct effects on online evaluation found in the regression models. These are hypotheses 4a and 4c and hypotheses 5a and 5c.

## Discussion and conclusion

Our study had two primary objectives. First, collecting students’ evaluation of online teaching facets compared to classroom teaching and second, explaining the role of study restrictions, communicative resources and academic exhaustion in overall evaluations of students.

Before discussing our findings, we want to address the limitations of our study. The design of student surveys is always a trade-off between short and straightforward responses on the one hand and scientifically sound coverage of the constructs of interest on the other hand. With these diverging interests, we have to consider some typical limitations. First, the study is designed as a cross-sectional study, which does not allow for empirical testing of cause-effect relationships; the direction of causality can only be argued theoretically. Second, relationships between constructs may be biased if measured with the same method (Podsakoff and Organ 1986). This common method bias is a general issue for self-report surveys (Jordan and Troth 2020). The analysed constructs were surveyed at the largest possible distance within the survey to reduce common method bias. Additionally, the response scales were designed differently for the single constructs: Teaching evaluation used a slide bar compared to a Likert scale for academic exhaustion and multiple dichotomous variables for the study restriction index. According to Harman’s one-factor test (Total variance = 43.47%), common method bias is not an issue with our dataset, but it is also not that far from the threshold of 50%. Third, we cannot exclude a sampling bias in the voluntary participation in our study. We achieved a satisfactory response rate, and our sample roughly corresponds to the population of political science students at Kiel University in terms of age, gender, and course of study. However, it would not be surprising if those students with pronounced exhaustion did not participate in the survey.

Regarding the first of our objectives, the sub-facets of online teaching evaluations, we can conclude that, on average, students who have experienced university teaching both face-to-face and online prefer face-to-face teaching in all surveyed aspects. This finding applies equally to lectures and seminars, although these two teaching formats are different. One possible explanation is that, despite all efforts, emergency remote learning has not yet been fully overcome, or genuine online learning has not yet been fully achieved. Such a transformation of university teaching is particularly challenging at German universities because online teaching had no tradition before the pandemic, and lecturers still have little experience with online didactics. In our case, the university provided many workshops to train digital competence, and also the Social Science Institute made every effort to facilitate and promote good online teaching. Furthermore, the lecturers individually discussed and experimented with different teaching methods. Students noticed these efforts and often expressed their gratitude in the open comment fields of the questionnaire. Nevertheless, lecturers have no significant long-term experience and routines with digital solutions, online didactics and teaching methods. In addition, the students have no prior experience except the emergency remote learning experience, and it is known from existing research that previous experience enhances students learning strategies when taking online courses and improves the acceptance and evaluation of this form of teaching (Wang et al. 2020; Haverila 2011; Wang et al. 2013). Therefore, the reason for the negative evaluation may be the as yet unrealised potential and the comparison to a long-established tradition of face-to-face teaching. In addition, students have not voluntarily chosen online teaching, which can cause reactance and subsequently a negative attitude. We consider supportive measures in the form of didactic training or peer-to-peer coaching among lecturers in order to benefit the perceived quality of online teaching.

With this explanation for the overall level of evaluation in mind, we can now look at the sub-facets. The students’ preference for classroom teaching is particularly evident in the aspects of concentration and attention in lectures and seminars, as well as in the quality of discussions in seminars. These results are in line with the findings of previous studies and possess a major challenge for online teaching in political science. Lack of concentration can be caused by even less physical activity in front of the screen compared to attending in person. There are many low-threshold distractions while working at home, e.g. through email and the internet or generally through the living situation. In lectures, asynchronous offers could help to increase attention, as the free choice of time should reduce distractions. A higher frequency in the alternation of teaching methods and the promotion of direct address and dialogue could help against concentration difficulties. The poor assessment of discussion quality in online seminars is of profound significance for a subject such as political science, which thrives on lively seminar discussions. Analytical and critical thinking, and subsequently, a certain logic of argumentation, constitute an inherent part of political science and an important learning objective for students. Such skills are primarily imparted through seminar discussions.

The degree to which students favour face-to-face teaching depends on their study constraints and the extent of interaction with other students. The influence of all variables is more profound on seminar evaluation than on lecture evaluation. This result is not surprising, as online seminars place higher demands on students’ technical and spatial equipment and are designed more for professional and social exchange with fellow students.

Academic exhaustion fully mediates the impact of study constraints on teaching evaluation and partly mediates the effect of social exchange. The full mediation path is in line with our assumption that constraints can be considered a study demand with related psychological costs leading to exhaustion, which is attributed to the teaching offer. The partial mediation in the relationship between social exchange and evaluation can potentially be explained by distinguishing two distinct kinds of communication. Professional on-topic exchange with fellow students and an off-topic exchange, which entails getting to know each other, small-talk, etc. We assume that the missing social component leads to exhaustion with a negative effect on evaluation in turn. The quality of professional exchange is directly associated with the teaching quality, as indicated by the remaining direct path to teaching evaluation in our mediation model.

This distinction is also relevant with regard to undergraduate students at the beginning of their studies, who were not included in the analysis due to the construction of the evaluation variables. Unlike students already studying before the pandemic began, they did not have the opportunity to form friendships and networks with other students. Among them, the feeling of isolation and insufficient contact with fellow students will be even more widespread, with corresponding impacts on the level of academic exhaustion and the evaluation of online teaching. The longer universities remain in digital-only mode, the more pressing this problem is likely to become.

Contrary to expectations, the contact and dialogue with lecturers do not influence online teaching evaluation when controlled for social exchange between students. Frisby et al. (2020) came to a comparable conclusion regarding the importance of the relationship with lecturers and fellow students, focusing on academic resilience and overcoming academic challenges. They also found no significant influence regarding lecturers when contact with fellow students was accounted for. However, it should not be inferred from this result that the role of the lecturers is not that important. For one thing, contact that is perceived as sufficient can significantly reduce students’ academic exhaustion. This is particularly important in times of corona pandemic, which are characterised by high levels of exhaustion among students. Furthermore, the results underline that lecturers should emphasise sufficient interaction between students when designing online classes. Activation and peer learning have to be encouraged. Various approaches are conceivable to achieve this goal. In addition to a more advisory role of the lecturer, activating teaching concepts are essential. One possibility would be the flipped classroom model, which has been successfully applied in seminars and political science lectures (Goerres et al. 2015; Whitman Cobb 2015; Bowers 2019) and has been shown to improve student learning and teaching evaluations in online courses in times of Covid-19 (Tang et al. 2020).

Beyond that, our results show that lecturers should pay more attention to the online discussion quality in their seminars and to the training of digital (discussion) competence among their students. Despite the respondents’ preference for face-to-face teaching, we do not assume its general superiority over online teaching. The evaluation took place in times of involuntary online-only teaching in the context of an uncertain pandemic situation. In the aftermath of the pandemic, we advocate a range of hybrid teaching formats as the new normal to create synergies between online and offline teaching and prepare students for a more digitalized working life.
